# LRIG2 expression and prognosis in non-small cell lung cancer

**DOI:** 10.3892/ol.2014.2157

**Published:** 2014-05-19

**Authors:** GUANGCHUAN WANG, JIE WU, HUIJUAN SONG

**Affiliations:** 1 Department of Immunology, Liaoning Medical University, Jinzhou, Liaoning 121000, P.R. China; 2 Department of Oncology, The First Affiliated Hospital of Liaoning Medical University, Jinzhou, Liaoning 121000, P.R. China; 3 Central Laboratory, Liaoning Medical University, Jinzhou, Liaoning 121000, P.R. China

**Keywords:** non-small cell lung cancer, immunohistochemistry, leucine-rich repeats and immunoglobulin-like domains 2, real-time polymerase chain reaction

## Abstract

The human leucine-rich repeats and immunoglobulin-like domains 2 (LRIG2) protein has been shown to be of prognostic value in several types of human cancer, however, the expression profiles of LRIG2 have not been described in non-small cell lung cancer (NSCLC). The present study evaluated the mRNA expression of LRIG2 in tumor specimens obtained from 39 NSCLC patients by SYBR Green quantitative polymerase chain reaction and the protein expression of LRIG2 in formalin-fixed paraffin sections obtained from 116 NSCLC patients by immunohistochemistry. The correlations between LRIG2 expression and clinicopathological data were analyzed. The patient survival data were collected retrospectively and the possible prognostic value of LRIG2 protein expression was investigated. The results showed that the mRNA expression of LRIG2 was decreased in NSCLC cancer tissues, which was associated with histological subtypes and tumor differentiation status. The protein expression of LRIG2 was only observed in the cytoplasm of the tumor tissue, which conformed to the mRNA expression results. Furthermore, the patients with high LRIG2 cytoplasmic expression showed poor survival times, and the five-year survival rate for patients with high LRIG2 expression was 27.8%, compared with 38.8% for patients with low expression (P=0.034), indicating that LRIG2 expression levels may have a potential role in the pathogenesis of NSCLC, and also a significant prognostic value. Further studies are required to fully elucidate the exact function of LRIG2 in NSCLC.

## Introduction

Lung cancer is the most frequently diagnosed cancer and the leading cause of cancer-related mortality in economically developed and developing countries (1,2). In addition, lung cancer was predicted to represent 26% of all female and 29% of all male cancer-related mortalities in 2012 (3). Non-small cell lung cancer (NSCLC) is the most common histological type, accounting for ~85% of lung cancer diagnoses in the USA (4). The most common forms of NSCLC include adenocarcinoma (AC), squamous cell carcinoma (SCC) and large cell carcinoma (5). Despite recent advances in the treatment of lung cancer, the overall five-year survival rate for such tumors remains poor at <15% (3). Thus, in order to develop rational and targeted therapies for NSCLC, an improved understanding of the molecular etiology of these tumors is required.

The human leucine-rich repeats and immunoglobulin-like domains (LRIG) gene family is comprised of three members, which are located at chromosome bands 3p14.3 (LRIG1), 1p13 (LRIG2) and 12q13 (LRIG3) (6–8). The LRIG genes encode integral membrane proteins consisting of a signal peptide, a leucine-rich repeat domain, a transmembrane domain, three LRIGs and a cytoplasmic tail (7). Increasing evidence indicates that, in certain cancer types, LRIG1 is a tumor suppressor (9). For example, LRIG1 expression is decreased in renal cell carcinoma (10), while high LRIG1 expression is associated with an improved prognosis in breast cancer (11), early stage invasive squamous cervical cancer (12) and cutaneous SCC (13). By contrast, in oligodendroglioma and uterine cervical carcinoma patients, LRIG2 expression in the cytoplasm has been demonstrated to correlate with poor survival (14,15). LRIG3 may also have a similar tumor suppressive function compared with LRIG1 (16). Furthermore, the LRIG proteins may have different roles depending on their subcellular localization. LRIG1 has not been associated with improved survival when expressed in the perinulear region, while the protein expression of LRIG2 and LRIG3 in the perinuclear area of astrocytoma cells has been found to correlate with improved patient survival (17). However, the expression profiles of LRIG2 have not been described in NSCLC. In the present study, quantitative polymerase chain reaction (qPCR) method and immunohistochemistry (IHC) were used to detect the mRNA and protein expression status of LRIG2. In addition, the potential associations between LRIG2 protein expression in NSCLC and the histological subtypes, clinical stage, differentiation status and survival were analyzed.

## Materials and methods

### Sample collection

The study population included two groups. Firstly, for the qPCR detection of LRIG2 mRNA expression, 39 NSCLC tissues and matched paracancerous and normal tissues were collected from patients with NSCLC at the Department of Thoracic Surgery at the First Affiliated Hospital of Liaoning Medical University (Jinzhou, China) between May 2010 and August 2012. Patients provided written informed consent prior to the specimen collection. None of the patients had received chemotherapy, radiotherapy or immunotherapy prior to the surgery. All samples were randomly selected regardless of age, gender or duration of the disease, and all cases were diagnosed pathologically. The clinicopathological data were retrospectively collected by reviewing the patients’ medical charts. Secondly, 125 formalin-fixed paraffin-embedded tissues of NSCLC samples were obtained for the immunohistochemical analysis of LRIG2. Patients enrolled in the study were followed to obtain five-year survival data. Survival was defined as the time between the surgery of the primary tumor and mortality or final follow-up of the patient. Due to loss to follow-up, nine patients were excluded from the study. All cases were classified according to the World Health Organization (WHO) revised proposal for histological types of lung and pleural tumors (18) and the tumor-node-metastasis staging was performed according to the UICC 1997 criteria. The study was performed with respect to the ethical standards of the 1975 Declaration of Helsinki, as revised in 2000, and was approved by the Ethics Committee of Liaoning Medical University (China). The clinicopathological data are summarized in Table I.

### qPCR

Total RNA was extracted using the TRIzol RNA kit (Invitrogen Life Technologies, Carlsbad, CA, USA) according to the manufacturer’s instructions. The first-strand complementary DNA (cDNA) was prepared from total RNA using a first-strand PrimeScript™ RT reagent kit with gDNA Eraser (Takara Bio, Inc., Shiga, Japan). Next, 1 μg total RNA was used as a template for reverse transcription (RT). The RT reaction was performed under the conditions of 37°C for 15 min and 85°C for 5 sec, followed by 42°C for 2 min with the gDNA Eraser. The PCR was performed using a primer specific for LRIG2 and the housekeeping gene, glyceraldehyde-3-phosphate dehydrogenase (GAPDH), sequence. Primers spanning at least one intron were selected to minimize inaccuracies due to genomic DNA contamination. The following primer sequences were used: LRIG2 sense, 5′-TGTGCACACCCTGAATGGCTA-3′ and antisense, 5′-TGT GTCCTTATCTGTGGCTTGAGAA-3′ (PCR product length, 98 bp); and GAPDH sense, 5′-GCACCGTCAAGGCTGAGAAC-3′ and antisense 5′-TGGTGAAGACGCCAGTGGA-3′ (PCR product length, 138 bp).

The qPCR was run on a Mastercycler^®^ ep realplex (Eppendorf, Hamburg, Germany) using the SYBR^®^ Premix Ex Taq™ kit (Takara Bio, Inc.). Each reaction consisted of a 20-μl sample containing 2 μl cDNA, 0.2 μM of each primer and 10 μl 2X SYBR Premix Ex Taq. Each PCR also included a non-template negative control to check for primer-dimer. The cycling conditions were one cycle of denaturation at 95°C for 30 sec, followed by 40 three-segment cycles of amplification (95°C for 5 sec, 55°C for 30 sec and 72°C for 30 sec), where the fluorescence was automatically measured during PCR, and one three-segment cycle of product melting (95°C for 15 sec, 60°C for 15 sec and 95°C for 15 sec). The baseline adjustment method of the Mastercycler ep realplex (Eppendorf) software was used to determine the cycle threshold (C_T_) in each reaction. A melting curve was constructed for each primer pair to verify the presence of one gene-specific peak and the absence of primer dimer. All samples were amplified in triplicate and the mean was used for further analysis. The PCR products were electrophoresed on a 3% agarose gel stained with ethidium bromide, and calculations were made using the ΔΔC_T_ method, as previously described (19). GAPDH was used as an internal control gene in order to normalize the PCR for the amount of RNA added to the RT reactions.

### IHC

IHC was performed on the formalin-fixed paraffin sections. In brief, 5-μm sections were dewaxed, rehydrated and incubated in 0.3% (V/V) hydrogen peroxide in 0.01 M phosphate-buffered saline (pH 7.6) for 20 min to inactivate the endogenous peroxidase. Antigen retrieval was performed using 0.01 M sodium citrate buffer (pH 6.0) under high pressure for 2 min. Next, the sections were immunostained with 2 μg/ml anti-LRIG2 primary antibody (Abcam, Cambridge, UK) at 4°C overnight and then stained with a horseradish peroxidase/Fab polymer-conjugated secondary antibody (Beijing Zhongshan Golden Bridge Biotechnology Co., Ltd., Beijing, China) for 30 min at room temperature. Finally, the antibody was revealed by diaminobenzidine at room temperature for 1 min and counterstained with hematoxylin for 15 min. All sections were examined and scored independently by two investigators who were blinded to the clinical details, and at least five fields were randomly selected. The immunostaining was scored as previously described (15). The expression was scored as high when ≥50% of the cancer cells were immunopositive and as low when <50% of the cancer cells were immunopositive or negative. This cut-off was selected as it showed the best explanatory power of the various cut-offs tested (0, 20, 50 and 100%). The subcellular localization of the staining was also evaluated.

### Statistical analysis

All statistical analyses were performed using the SPSS 16.0 statistical software package (SPSS, Inc., Chicago, IL, USA). A two-sample t-test for independent samples and a χ^2^ test were used for continuous and categorical variables, respectively. The one-way analysis of variance was used to compare the means of two or more independent groups. The Kaplan-Meier estimator was used to calculate the survival rate probability as a function of time and the log-rank test was used to compare survival time between the groups. All statistical tests were two-sided and P<0.05 was considered to indicate a statistically significant difference.

## Results

### Correlation between LRIG2 mRNA expression status and clinicopathological variables

**Figure 1:**
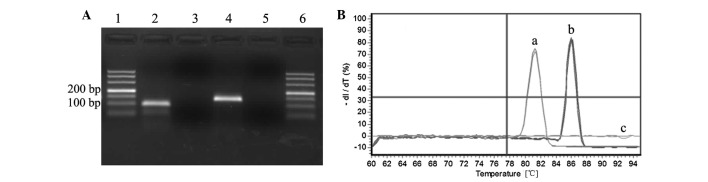
qPCR assay. (A) Gel electrophoresis analysis of the target and reference gene PCR products. Lanes 1 and 6, 500-bp molecular marker; 2, LRIG2; 3, NTC for LRIG2; 4, GAPDH; and 5, NTC for GAPDH. (B) Melting curve analysis of the target and reference gene PCR products. The SYBR Green qPCR reactions of the (a) LRIG2 and (b) GAPDH genes were performed using a normal sample of the complementary DNA (cDNA). Each peak corresponds to a unique PCR product. (c) NTC reactions showed no PCR product. LRIG2, leucine-rich repeats and immunoglobulin-like domains 2; NTC, non-template negative control; qPCR, quantitative polymerase chain reaction; GAPDH, glyceraldehyde-3-phosphate dehydrogenase; −dI/dT, negative first derivative of the melting curves as a function of temperature.

Melting curve analysis confirmed the specific amplification of the target and reference genes. Furthermore, the gel electrophoresis analysis of the amplification products revealed a single band with the predicted size for LRIG2 (98 bp) and GAPDH (138 bp) (Fig. 1). The slopes of the standard curves were −3.242 and −3.238 for the GAPDH and LRIG2 genes, respectively. The reliability of the PCR reaction efficiencies was also assessed by plotting ΔC_T_ values (C_T_ LRIG2 - C_T_ GAPDH), and the absolute value of the trend line slopes was ≤0.1, which indicated the validity of the relative quantitative assay by ΔΔC_T_ method.

The LRIG2 mRNA expression was examined in 39 pairs of NSCLC and adjacent cancerous tissues using the ΔΔC_T_ method. The results showed that the mRNA expression of LRIG2 was decreased in the cancer and adjacent cancerous tissues. The mean mRNA expression level of LRIG2 in the 39 NSCLC cancer and adjacent cancerous tissues was 0.2288±0.0230 and 0.6185±0.0321, respectively. The correlation between LRIG2 mRNA expression in the cancer tissues and the various clinicopathological parameters were further analyzed and are shown in Table II. The expression of LRIG2 mRNA was significantly higher in AC compared with that in SCC (P=0.005). According to the tumor differentiation status, a significant downregulation of LRIG2 (P=0.013) was also observed, while no significant correlation was observed between LRIG2 expression and tumor staging (P=0.822). In addition, no correlation was observed between LRIG2 mRNA expression and age, gender and smoking habits (data not shown). The correlation between LRIG2 mRNA expression in adjacent cancerous tissues and the various clinicopathological parameters were also analyzed. However, no correlations were identified between LRIG2 mRNA expression and the clinicopathological parameters.

### Correlation between LRIG2 protein expression and the clinicopathological features of NSCLC

**Figure 2:**
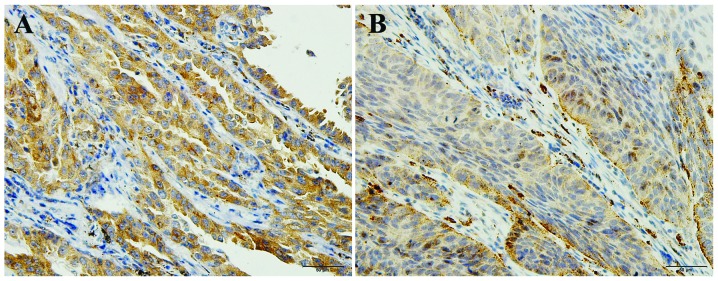
Immunostaining for leucine-rich repeats and immunoglobulin-like domains 2 (LRIG2) in the tissue samples of non-small cell lung cancer (NSCLC). (A) Adenocarcinoma (AC) and (B) squamous cell carcinoma (SCC) (magnification, ×400).

By IHC analysis, specific LRIG2 immunoreactivity was generally only observed in the cytoplasm (Fig. 2). According to the LRIG2 immunoreactive intensity, in the total 116 cases of NSCLC, 80 patients (68.97%) were classified into the low-LRIG2 group, and 36 (31.03%) were classified into the high-LRIG2 group. A statistically significant correlation was found between LRIG2 expression and the two major histological subtypes (AD and SCC; P=0.048), which also conformed to the results of the qPCR. The correlation between LRIG2 expression and the various clinicopathological parameters of 116 cases of NSCLC was also analyzed. Briefly, the LRIG2 staining level was found to significantly correlate with differentiation status (P=0.034), as shown in Table III. No significant correlation was identified between LRIG2 expression and patient gender, age, smoking history and tumor staging (all P>0.05).

### Correlation between LRIG2 protein expression and overall survival

**Figure 3:**
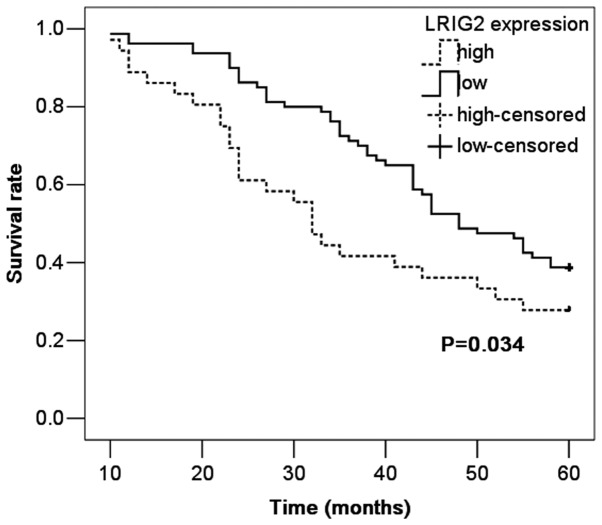
Kaplan-Meier curves showing the survival of non-small cell lung cancer (NSCLC) patients in relation to LRIG2 expression (log-rank test, P=0.034). LRIG2, leucine-rich repeats and immunoglobulin-like domains 2.

The prognostic value of LRIG2 protein expression for overall survival in NSCLC patients was evaluated by comparing the patients with high and low LRIG2 expression. According to the Kaplan-Meier survival analysis, the patients with high LRIG2 expression exhibited evidently lower overall survival rates than those with low LRIG2 expression (P=0.034; Fig. 3). The five-year survival rate for patients with high LRIG2 expression was 27.8%, compared with 38.8% for patients with low expression. Multivariate analysis was conducted using the Cox proportional hazards model to examine the impact of LRIG2 expression and other clinicopathological parameters, including tumor differentiation status and tumor stage. The expression of LRIG2 emerged as an independent and significant factor associated with poor five-year survival rates (P=0.019). Thus, LRIG2 expression levels may have a prognostic value in NSCLC patients.

## Discussion

LRIG2 is an integral membrane protein that is widely expressed in human tissues (7,8). Although LRIG2 has been proven to be of prognostic value in several types of human cancers (16), the expression status in NSCLC remains unknown. The current study provides the first characterization of the LRIG2 expression status in human NSCLC. It was found that the expression of LRIG2 was decreased in the cancer tissues, indicating a potential role of the LRIG2 protein in the pathogenesis of NSCLC. In addition, the cytoplasmic expression level of LRIG2 was associated with poor prognosis, suggesting that LRIG2 may have prognostic value in NSCLC patients. These conclusions were consistent with the results obtained in other types of human cancer (14,15).

LRIG2 expression has been investigated in several types of cancer. Guo *et al* (17) found that perinuclear staining of LRIG2 was associated with a low WHO grade in astrocytic tumors, and compared with the normal pituitary samples, the expression of LRIG2 was lower in the human pituitary adenoma HP75 cell line. Wang *et al* (20) also described the consequences of selectively knocking down LRIG2 expression in the glioma GL-15 cell line. The study found that the downregulation of LRIG2 expression decreased the proliferation rate, which resulted in G_0_/G_1_ arrest and the increased spontaneous apoptosis, cell adhesion and invasion capability of the glioma cell line. In addition, in cells lacking LRIG2, the activation of ErbB1 (epidermal growth factor receptor) was reduced through increased ErbB1 degradation and decreased ErbB phosphorylation (20). LRIG2 expression was also found in the precancerous cervical epithelium and shown to increase with increasing cervical intraepithelial neoplasia grade (21). An association was also found between the expression of LRIG2 and specific tumor markers. Similarly, LRIG2 expression was found to correlate with increased FHIT and p16^4INKa^, as well as IL-10 expression, while a negative correlation was observed with Rb and Ki-67 expression (21). In meningiomas, the LRIG2 expression in the cytoplasm of has been found to correlate with estrogen receptor (ER) status and histological subtype, with the benign subtypes most frequently expressing LRIG2 (22). Recently, emerging evidence has indicated that the hormones, estrogen and progesterone, are key in the progression of NSCLC (23). LRIG2 is a glycoprotein with N-linked oligosaccharides, and a recent study found that LRIG2 has a physical association with FBXO6 (also known as FBX6) (24), which is involved in the endoplasmic reticulum-associated degradation pathway by mediating the ubiquitination of glycoproteins. The F box protein, Fbx6, also regulates Chk1 (a key protein kinase in the replication checkpoint) stability and cellular sensitivity to replication stress (25). In the present study, the mRNA expression of LRIG2 was decreased in NSCLC cancer tissues and found to correlate with histological subtypes and tumor differentiation status. The protein expression of LRIG2 also conformed to the mRNA expression results. This indicates a potential role for LRIG2, which may interact with ER and FBX6 in the pathogenesis of NSCLC. However, further study is required to confirm this hypothesis.

The LRIG2 protein localizes to different subcellular compartments, including the nucleus, perinuclear area, cytoplasm and cell surface (9). The subcellular localization of LRIG2 also appears clinically important. Hedman *et al* (15) found that high LRIG2 expression correlates with poor survival in invasive early-stage squamous cervical cancer. In addition, Holmlund *et al* (14) reported that the expression of cytoplasmic LRIG2 is a negative prognostic factor for oligodendroglioma. As for pituitary adenoma, LRIG2 expression has been found to predict the invasiveness of pituitary tumors and a poor prognosis (26). In esophageal carcinoma, a trend towards decreased survival was found for the high expression of LRIG2, however, this trend was not statistically significant (27). By contrast, the protein expression of LRIG2 in the perinuclear area of astrocytoma cells has been found to correlate with improved patient survival (17). In the present study, only the cytoplasmic expression of LRIG2 was observed and the patients with high LRIG2 expression were associated with poor survival, which is consistent with the results of several other studies (14,15). Certain studies (28,29) have indicated that the LRIG2 protein exhibits different roles in human tumors depending on their subcellular localization. LRIG2 localization in the cytoplasm may augment its action as a tumor promoter, whereas the perinuclear localization of LRIG2 may act as a tumor suppressor. The significance of the specific subcellular localization of LRIG2 protein requires further investigation, as well as the mechanism of its different functions.

In conclusion, the present study showed that LRIG2 expression is decreased in NSCLC tissues, which indicates a potential role in the pathogenesis of NSCLC. In addition, the cytoplasmic expression of LRIG2 was found to be an independent prognostic factor associated with poor survival in NSCLC. This may have prognostic value in NSCLC patients. However, to fully elucidate the exact function of LRIG2 in NSCLC, further and larger studies are required.

## Figures and Tables

**Table I:** Characterization of the NSCLC patients included in the study.

	Patients, n	
	
Clinicopathological features	qPCR analysis (n=39)	IHC analysis (n=116)
Gender, female/male	14/25	45/71
Median age at diagnosis, years (range)		
Female	60.5 (44–71)	59.5 (42–70)
Male	60.0 (39–79)	57.5 (38–79)
Histological subtypes		
Adenocarcinoma	21	86
Squamous cell carcinoma	18	30
Differentiation status		
Well	14	45
Moderate	12	47
Poor	13	24
Tumor staging		
IA-IB	15	48
IIA-IIB	16	44
IIIA	8	24

NSCLC, non-small cell lung cancer; qPCR, quantitative polymerase chain reaction; IHC, immunohistochemisty.

**Table II:** Correlation between LRIG2 mRNA expression status and the clinicopathological features of patients.

		LRIG2 expression, mean ± SEM			
		
Variables	Patients, n	Cancer tissues	P-value	Adjacent to cancer tissues	P-value
Histological subtypes					
Adenocarcinoma	21	0.2869±0.0318	0.005^a^	0.6744±0.0396	0.059^a^
Squamous cell carcinoma	18	0.1609±0.0258		0.5533±0.0489	
Differentiation status					
Well	14	0.3140±0.0424	0.013^b^	0.6695±0.0478	0.488^b^
Moderate	12	0.2012±0.0302		0.6011±0.0534	
Poor	13	0.1625±0.0327		0.5795±0.0659	
Tumor staging					
IA-IB	15	0.2430±0.0398	0.822^b^	0.5808±0.0595	0.228^b^
IIA-IIB	16	0.2113±0.0351		0.6845±0.0424	
IIIA	8	0.2370±0.0507		0.5573±0.0651	

Calculated by ^a^independent samples t-test and ^b^one-way analysis of variance. LRIG2, leucine-rich repeats and immunoglobulin-like domains 2; SEM, standard error of the mean.

**Table III:** Correlation between LRIG2 protein expression status and the clinicopathological features of patients.

		LRIG2 expression, n		
			
Clinicopathological features	Patients, n	High score	Low score	P-value
Histological subtypes				
Adenocarcinoma	86	31	55	0.048
Squamous cell carcinoma	30	5	25	
Differentiation status				
Well	45	20	25	0.034
Moderate	47	12	35	
Poor	24	4	20	
Tumor staging				
IA-IB	48	15	33	0.948
IIA-IIB	44	13	31	
IIIA	24	8	16	

LRIG2, leucine-rich repeats and immunoglobulin-like domains 2.

## References

[1] Jemal A, Bray F, Center MM, Ferlay J, Ward E, Forman D (2011). Global cancer statistics. CA Cancer J Clin.

[2] Saintigny P, Burger JA (2012). Recent advances in non-small cell lung cancer biology and clinical management. Discov Med.

[3] Siegel R, Naishadham D, Jemal A (2012). Cancer statistics, 2012. CA Cancer J Clin.

[4] Molina JR, Yang P, Cassivi SD, Schild SE, Adjei AA (2008). Non-small cell lung cancer: epidemiology, risk factors, treatment, and survivorship. Mayo Clin Proc.

[5] Collins LG, Haines C, Perkel R, Enck RE (2007). Lung cancer: diagnosis and management. Am Fam Physician.

[6] Nilsson J, Vallbo C, Guo D (2001). Cloning, characterization, and expression of human LIG1. Biochem Biophys Res Commun.

[7] Holmlund C, Nilsson J, Guo D (2004). Characterization and tissue-specific expression of human LRIG2. Gene.

[8] Guo D, Holmlund C, Henriksson R, Hedman H (2004). The LRIG gene family has three vertebrate paralogs widely expressed in human and mouse tissues and a homolog in Ascidiacea. Genomics.

[9] Hedman H, Henriksson R (2007). LRIG inhibitors of growth factor signalling - double-edged swords in human cancer?. Eur J Cancer.

[10] Thomasson M, Hedman H, Guo D, Ljungberg B, Henriksson R (2003). LRIG1 and epidermal growth factor receptor in renal cell carcinoma: A quantitative RT-PCR and immunohistochemical analysis. Br J Cancer.

[11] Krig SR, Frietze S, Simion C (2011). Lrig1 is an estrogen-regulated growth suppressor and correlates with longer relapse-free survival in ERalpha-positive breast cancer. Mol Cancer Res.

[12] Lindstrom AK, Ekman K, Stendahl U (2008). LRIG1 and squamous epithelial uterine cervical cancer: correlation to prognosis, other tumor markers, sex steroid hormones, and smoking. Int J Gynecol Cancer.

[13] Tanemura A, Nagasawa T, Inui S, Itami S (2005). LRIG-1 provides a novel prognostic predictor in squamous cell carcinoma of the skin: immunohistochemical analysis for 38 cases. Dermatol Surg.

[14] Holmlund C, Haapasalo H, Yi W (2009). Cytoplasmic LRIG2 expression is associated with poor oligodendroglioma patient survival. Neuropathology.

[15] Hedman H, Lindstrom AK, Tot T, Stendahl U, Henriksson R, Hellberg D (2010). LRIG2 in contrast to LRIG1 predicts poor survival in early-stage squamous cell carcinoma of the uterine cervix. Acta Oncol.

[16] Muller S, Lindquist D, Kanter L (2013). Expression of LRIG1 and LRIG3 correlates with human papillomavirus status and patient survival in cervical adenocarcinoma. Int J Oncol.

[17] Guo D, Nilsson J, Haapasalo H (2006). Perinuclear leucine-rich repeats and immunoglobulin-like domain proteins (LRIG1–3) as prognostic indicators in astrocytic tumors. Acta Neuropathol.

[18] Shimosato Y (2002). Histological typing of lung and pleural tumors (3rd edition, 1999): Malignant epithelial tumors. Nihon Rinsho.

[19] Kontos CK, Papadopoulos IN, Fragoulis EG, Scorilas A (2010). Quantitative expression analysis and prognostic significance of L-DOPA decarboxylase in colorectal adenocarcinoma. Br J Cancer.

[20] Wang B, Han L, Chen R (2009). Downregulation of LRIG2 expression by RNA interference inhibits glioblastoma cell (GL15) growth, causes cell cycle redistribution, increases cell apoptosis and enhances cell adhesion and invasion *in vitro*. Cancer Biol Ther.

[21] Lindstrom AK, Asplund A, Hellberg D (2011). Correlation between LRIG1 and LRIG2 expressions and expression of 11 tumor markers, with special reference to tumor suppressors, in CIN and normal cervical epithelium. Gynecol Oncol.

[22] Ghasimi S, Haapasalo H, Eray M (2012). Immunohistochemical analysis of LRIG proteins in meningiomas: correlation between estrogen receptor status and LRIG expression. J Neurooncol.

[23] Kazmi N, Marquez-Garban DC, Aivazyan L (2012). The role of estrogen, progesterone and aromatase in human non-small-cell lung cancer. Lung Cancer Manag.

[24] Liu B, Zheng Y, Wang TD (2012). Proteomic identification of common SCF ubiquitin ligase FBXO6-interacting glycoproteins in three kinds of cells. J Proteome Res.

[25] Zhang YW, Brognard J, Coughlin C (2009). The F box protein Fbx6 regulates Chk1 stability and cellular sensitivity to replication stress. Mol Cell.

[26] Zhang H, Yan Q, Xu S (2011). Association of expression of Leucine-rich repeats and immunoglobulin-like domains 2 gene with invasiveness of pituitary adenoma. J Huazhong Univ Sci Technolog Med Sci.

[27] Wu X, Hedman H, Bergqvist M (2012). Expression of EGFR and LRIG proteins in oesophageal carcinoma with emphasis on patient survival and cellular chemosensitivity. Acta Oncol.

[28] Musacchio M, Perrimon N (1996). The Drosophila kekkon genes: novel members of both the leucine-rich repeat and immunoglobulin superfamilies expressed in the CNS. Dev Biol.

[29] Eisenstat DD, Gibson SB (2009). RIGging functional outcomes in glioma cells: new insights into LRIG proteins in malignant gliomas. Cancer Biol Ther.

